# Sex Differences in Lifestyle Behaviors among U.S. College Freshmen

**DOI:** 10.3390/ijerph16030482

**Published:** 2019-02-07

**Authors:** Melissa D. Olfert, Makenzie L. Barr, Camille C. Charlier, Geoffrey W. Greene, Wenjun Zhou, Sarah E. Colby

**Affiliations:** 1 Davis College of Agriculture, Natural Resources and Design, Division of Animal & Nutritional Sciences, West Virginia University, Morgantown, WV 26506, USA; mbarr6@mix.wvu.edu; 2 Clinical & Translational Science, West Virginia University, Morgantown, WV 26506, USA; ccharlie@hsc.wvu.edu; 3 Department of Nutrition and Food Sciences, 125 Fogarty Hall, University of Rhode Island, Kingston, RI 02881, USA; gwg@uri.edu; 4 Department of Business Analytics and Statistics, University of Tennessee, Knoxville, TN 37996, USA; wzhou4@utk.edu; 5 Department of Nutrition, University of Tennessee, Knoxville, TN 37996, USA; scolby1@utk.edu

**Keywords:** sex differences, health behaviors, self-report, freshmen, college

## Abstract

Within lifestyle behavior research, the sex of populations causes differences in behaviors and outcomes of studies. This cross-sectional study investigated lifestyle behavior patterns in college students, examining sex differences in four areas: Nutrition, physical activity, sleep, and stress. Data from over 1100 college freshmen across 8 United States universities were used for this cross-sectional analysis. Self-reported data assessed fruit and vegetable intake, fat percent intake, physical activity, perceived stress, and sleep quality. Statistical analysis included Pearson chi-squared and Mann–Whitney’s U tests for scores by sex. Likewise, healthy cut-offs were used to determine frequency of participants within range of the five tools. Males reported higher intake of both fruits and vegetables, and percent energy from fat than females. Males also reported higher physical activity levels, lower stress levels, and poorer sleep quality than females. Of the five self-reported tools, males were found to have a larger frequency of participants with healthy ranges than females. In a large college freshmen sample, sex was found to be related to general lifestyle behaviors which strengthen results reported in the previous literature. These findings shed light on the need for lifestyle behavior interventions among at-risk college students to enhance their behaviors to healthy levels.

## 1. Introduction

Among lifestyle behavior research, the intersecting relationship of nutrition, physical activity, sleep, and stress is influential in the lives of young adults whether while in schooling or vacation [1,2]. As studies investigating these issues in young adults are becoming more frequent, tailored interventions and programming are emerging. Additionally, there has been a call to the research for lifestyle interventions at college as chronic diseases are now becoming prevalent in our younger generations earlier in life [3].

Specifically, when looking at health behaviors, sex differences have played a role in differences in behaviors. We do find that literature suggests targeting specific behaviors for males and female college students separately [4]. However, with limited services on campus for weight-management-related health programs and a large focus on sexual health and alcohol abuse education, the need for targeted health and weight interventions is warranted [5].

Between the different sexes, men appear to be less interested in learning about wellness and display lower nutritional knowledge than women [6,7]. Men have also been found to be less likely to believe that they are knowledgeable of dietary guidelines, although both sexes reported being confident about knowing what constitutes healthy nutrition in general [8]. Perhaps as a result, men tend to report lower fruit and vegetable serving intake along with higher fat consumption than women [9,10]. Men also typically prefer more traditional food (i.e., meals comprised of meat, potatoes, and vegetables) [8]. Women’s nutritional behaviors appear to be more intentionally influenced by weight concerns [9] than those of their male counterparts and often include experimentation with various types of diets, including low-fat and low-carbohydrate plans [6]. In the college student population, it is important to note that both males and females tend to display an overall adequate knowledge of what constitutes a healthy diet [7], thereby suggesting that differences in food consumption across sex are not exclusively a result of lack of nutrition education, although the latter should remain a vital component of health promotion on campus [11] as intentions and self-efficacy can play a role [12].

Although young adults traditionally tend to be relatively healthy, other lifestyle behaviors in addition to diet fall short of meeting national guidelines. Rates of physical activity in this group do not meet national recommendations that individuals 18–64 years old complete at least 150 minutes of moderate, or 75 minutes of vigorous, aerobic activity a week [13,14]. According to the American College Health Association (ACHA), less than half of U.S. college students meet federal guidelines for aerobic physical activity [15] despite the well-established relationship between physical activity and academic success [16,17]. However, we do find males are more apt to perform more physical activity [18]. In addition, exercise has been found to reduce the effect of perceived stress during examination periods [19]. As college years play a significant role in lifelong lifestyle patterns, including physical activity, it is important to increase physical activity in college students [20].

Sex differences may also be associated with sleep patterns and overall quality of sleep. Males are 40% less likely to experience insomnia over the course of their lifetime than females [21]. Some of these differences have been attributed to ovarian steroid production in females, who tend to report poorer sleep in terms of quality, quantity, and interruption [21]. Male testosterone production appears to be positively linked to sleep cycles, including rapid-eye movement (REM) phases [21]. Young adults frequently experience poor sleep, with only approximately a third of college students reporting good sleep quality using the Pittsburgh sleep quality index (PSQI) scale and 29.4% reporting getting the 8 or more hours of sleep recommended for this population [22]. In college students, sleep quality has also been linked to stress, with personal and academic stress identified as negatively affecting sleep [22]. As with the general population, sex has been found to be a factor in college students’ sleep patterns with men experiencing better sleep quality [23,24]. In addition to sleep, college years have been identified as a stressful time for both sexes [25], and female college students have been found to have higher perceived stress levels than their male counterparts despite a higher awareness of general health habits [26,27]. With both stress and sleep quality playing a role in lifestyle behaviors and health, it is important to target all of these coexisting behaviors that are shown to influence quality of life [28]. Within behavioral interventions on college campuses, sex differences arise during post-intervention analysis; however, targeting these specific differences during interventions is limited when on a large-scale college student cohort. 

The goal of this study was to investigate sex differences in four main lifestyle behavior areas, nutrition, physical activity, sleep, and stress, and the proportion of students meeting healthy cut-off ranges in a large sample of college students across the United States. We hypothesized that females would have better self-reported health behaviors and less than half of participants would achieve 3 or more healthy categories. 

## 2. Methods

College freshmen students across 8 U.S. universities (Auburn University, Kansas State University, South Dakota State University, Syracuse University, University of Florida, University of Maine, University of Tennessee Knoxville, and West Virginia University) participated in the U.S. Department of Agriculture (USDA)-funded Get Fruved study. The Get Fruved study aimed to improve lifestyle behaviors among college students at four intervention schools compared to four control schools. The analysis described in this paper is based on a cross-sectional examination of baseline data from the Get Fruved study including all 8 schools. Incoming freshmen at each university were eligible to participate in the Get Fruved study if they screened “at-risk” based on a self-report screener. At a goal of 350 first-year students per site (total *n* = 2800), students were equally recruited at university orientation events, through parent newsletters, word-of-mouth, and during on-campus events to assist with selection bias. Inclusion criteria of being “at-risk” included self-reporting consuming less than 2 cups of fruits or 3 cups of vegetables per day and/or met one of the following: BMI ≥25 kg/m^2^, first-generation college student in family, overweight or obese parent, low affluence (i.e., low number of computers/laptops in household, not having own bedroom, low family travel frequency, and limited number of cars in family), or being a racial minority. Eligible participants were invited to complete an in-person assessment, including an in-depth behavioral survey. Final participants attending the in-person assessment who provided informed consent were an estimated portion of all eligible participants and were included in the current study sample. Data collection took place in August of 2015, during the students’ first semester on campus.

Trained researchers performed anthropometric measures at each assessment. Height was measured by standard stadiometer (Heightonic digital stadiometer; Issaquah, WA, USA) with the participant standing, facing forward without shoes. Weight was taken without shoes and with light clothing via digital scale (Electronic Tanita scale; Arlington Heights, IL, USA). All measures were taken twice and averaged. Repeated measures were taken a third time if initial measures had a range larger than 0.2 kilogram for weight and 0.2 centimeters for height. BMI was calculated as weight in kilograms divided by height in meters squared. 

Instruments used to assess behavior in the survey included five tools commonly used and validated in college populations, the National Cancer Institute fruit and vegetable screener (NCI FV) [29,30], the National Cancer Institute fat screener (NCI Fat) [31,32,33], the short-form international physical activity questionnaire (IPAQ) [34,35], Cohen’s perceived stress scale (PSS) [36,37], and the Pittsburgh sleep quality index (PSQI) [24]. The NCI FV is a 19-item survey assessing frequency and quantity of fruits and vegetables consumed over the last month reported as cup equivalents per day [30]. The NCI fat screener is a 16-item tool estimating daily percent energy from fat from a variety of sources, i.e., dairy, meat, oil, etc., over the last 12 months [38]. The PSQI is designed to evaluate sleep in the last month, with continuous scoring ranging between 0 to 21 with scores above 5.0 indicative of poor sleep quality [39]. The IPAQ was used to assess physical activity in metabolic equivalency tasks–minute/week (METs) over the previous month to give a total score of total physical activity, including walking, moderate, and vigorous activity [40]. Lastly, the PSS tool examines 14 self-reported questions regarding an individual’s stress over the last month and is scored on a scale of 0–56 with scores between 0–13 being low stress, 14–26 being moderate stress, and 27–40 representing high stress [41].

Initial statistical analyses included Pearson chi-squared test to examine categorical variable (demographics) relationships and Mann–Whitney’s U test to examine continuous variables (height, weight, BMI) by group (i.e., male and female). Nonparametric tests were used due to lack of normality of continuous variables. Analysis of covariance (ANCOVA) models were used to test self-reported behavioral screeners (NCI FV, NCI fat, IPAQ, PSQI, and PSS) by sex, controlling for BMI and age. Scores were also used to determine cut-offs of ‘healthy’ vs. ‘unhealthy’ scores for each variable on a dichotomous 0 (did not meet healthy cut-off) or 1 (met healthy cut-off) scale. A healthy score was determined by the following cut-offs for each tool: NCI FV of >5 cup servings/day, NCI fat percentage of <35.0%, an IPAQ score of >2295, PSS of <27, and PSQI <5. These variables were then summed to create a ‘healthy’ score of the 5 variables on a 0–5 scale. Pearson chi-square analysis was used to examine the relationship of healthy score categories by sex. Fisher’s exact test was used if cell sizes were below 5. One participant was self-reported as a zero on the ‘healthy score’ and was excluded from analysis to maintain anonymity. JMP version 13 software was used to perform the analysis [42].

The multistate umbrella Institutional Review Board (IRB) at University of Tennessee, Knoxville approved the study protocol for the University of Tennessee, West Virginia University, and South Dakota State University (IRB approval #14-09366 B-XP). The University of Florida IRB approved the same strategies for activities at the University of Florida (IRB approval #2014-U-0547FRUVED). The IRB approved study procedures for the control universities, Syracuse University and the University of Maine (#14-175 and #2014-06-21, respectively). This study was prospectively registered on clinicaltrials.gov, NCT02941497. All procedures were carried out following rules and procedures of the Declaration of Helsinki.

## 3. Results

### 3.1. Demographic Characteristics of Sample

Of the 5413 first-year students that had taken the eligibility screener (17% response rate per total first-year students), 2750 were deemed eligible to participate in the full in-person assessment. Those completing the in-person assessment were 1149 subjects from eight states. From this group, 1132 participants self-identified as either male or female (Table 1). Those excluded from the study sample included 19 subjects who did not complete demographic questions and 15 who chose not to answer the sex question. The sample was composed of two thirds female subjects (*n* = 749, 66.2%) and one third male subjects (*n* = 383, 33.8%). 

Of those completing the race/ethnicity question, the sample was predominantly non-Hispanic White only (*n* = 603), Hispanic/Latino (*n* = 203), Other (including biracial) (*n* = 190), and Black only (*n* = 117). Table 1 indicates demographic variables by sex. The population was predominately 18 years old (*n* = 968), but there were significant age differences between males and females (*p* < 0.01).

Mann–Whitney’s U test was used for testing gender differences in continuous variables (i.e., height, weight, BMI) shown in Table 2. The average height of the male group was 176.01 ± 7.46 cm and that of the female group was 164.28 ± 6.79 cm (*p* < 0.0001). The average weight of males was 76.24 ± 14.98 kg and that of females was 65.80 ± 15.24 kg (*p* < 0.0001). The average BMI of males was 24.56 ± 4.34 kg/m^2^ and that of females was 24.31 ± 5.08 kg/m^2^ (*p* = 0.0516).

### 3.2. Self-Reported Behaviors

An ANCOVA model was used to examine the relationship of behavioral screeners by sex while controlling for age and BMI. The mean NCI FV cup servings/day was 2.79 ± 2.70 for males and 2.27 ± 1.88 for females, while the mean NCI fat score was 30.01 ± 2.27 percent energy intake fat for males and 29.34 ± 2.22 percent energy fat for females. These scores are indicative of female students reporting a lower intake of fruit and vegetables and lower energy-adjusted fat intake than their male counterparts (*p* = 0.0011 and *p* < 0.0001, respectively) (Table 3). In both FV and fat, neither age nor BMI significantly contributed to the model.

Mean IPAQ MET min/week was statistically higher for males than for females when controlling for age and BMI (nonsignificant in model), at 3345.70 ± 2358.71 METs versus 2420.88 ± 1746.22 METs (*p* < 0.0001). Mean stress score for males was significantly lower than for females at 24.68 ± 6.02 versus 27.23 ± 5.91 (*p* < 0.0001), indicative of lower self-reported stress levels in male students than in their female counterparts when controlling for age and BMI (nonsignificant in model). Finally, males had a statistically significant lower mean PSQI score than females, at 5.59 ± 2.48 versus 5.95 ± 2.64 (*p* = 0.0485), indicative of better self-reported sleep quality in males than in females. When controlling for age and BMI in the PSQI ANCOVA model, age was not significant; however, BMI was a significant covariate (0.0442). 

When examining ‘healthy’ cut-off ranges for all utilized tools, Table 4 shows the frequency of participants being placed in healthy ranges (met cut-off values for healthy ranges). For healthy ranges of physically measured BMI (adults 18.5–24.9), there were no significant differences in the percentage of participants in healthy ranges by male or female. Males had slightly higher percentages falling within ‘healthy’ ranges of each self-reported behavior tool, except for NCI FV intake, when compared to females. However, those having significantly higher male presences were NCI FV (*p* = 0.0137), IPAQ (*p* < 0.0001), and PSS (*p* < 0.0001). The ‘healthy score’ (sum of each self-reported tool that met healthy cut-off range) is broken down into those meeting healthy range as a categorical score of 0–5. Pearson chi-squared analysis showed significant differences among score categories and sexes (*p* < 0.0001). Females had a larger proportion of participants reaching only two or three classifications of ‘healthy’ (30.5% and 29.9%, respectively), while males had larger percentages meeting the three and four cut-off classifications (30.7% and 28.7%, respectively).

## 4. Discussion

Results from this study reinforce the importance of considering the potential role of sex in key lifestyle behavior categories during college, as sex differences were found in all four areas investigated: Nutrition, physical activity, sleep, and stress. 

Nutritional intake was perhaps the most surprising category, as males reported a higher intake of percentage of energy from both fat and cup servings of fruit and vegetable. Both were consistent with the literature indicating a lower energy-adjusted dietary quality for males than for females; the higher absolute intake of fruits and vegetables in males can be attributed to higher overall energy intake of males compared to females [9,10]. Women were found to have the higher score with stress when compared to their male counterparts. Furthermore, female students were also found to experience lower sleep quality than male students, although both sexes had mean PSQI scores above the 5-point cut off indicative of poor sleep. As such, this sample’s sleep patterns appear to be similar to that previously observed in college students [22]. Findings of increased stress and decreased sleep quality in females were consistent with literature, both in the general population [2,21] and in younger demographics [22,27]. Findings from this sample support previous literature that found college males report higher levels of physical activity than college females [20]. This has additionally been backed through accelerometer data of males participating in more activity than females [43]. As these findings were at baseline when first-year students arrived on campus, moving forward with interventions to explain and educate on the importance of physical activity will be a vital factor [44,45]. Additionally, the link between the results in decreased stress levels and better sleep quality observed in males should likely be investigated further, as physical activity, sleep, and stress and known to be intricately related [46,47,48]. 

When further examining these self-reported measures, ‘healthy’ ranges for the tools were determined from previous literature and the scoring of each tool [30,38,39,40,41] along with healthy ranges of BMI. For healthy cut-off ranges, it was found that participants were predominantly classified as ‘healthy’ within only 3 of the 6 self-reported tools. When examined closer, male participants fell largely into the 3–4 healthy cut-off range of the tools, while females were among the 2–3 healthy cut-off range of the tools. These tools taken separately show that most participants are not meeting fruit and vegetable intake recommendations but are largely in the healthy range of fat intake. Likewise, nearly half of females and over half of males are in healthy ranges for physical activity and stress but below adequate in sleep quality. These findings suggest that interest in fruit and vegetable intake as well as sleep quality may be important to incoming freshmen students on college campuses. 

Although each self-reported survey tool was validated and reliable for the college population, some limitations of the current study are important to discuss. One limitation of this study may include documented difficulties in assessing true food consumption using self-reported behavior surveys [49,50]. Indeed, sex, race, and socioeconomic status have been found to affect instruments such as the NCI fat and the NCI FV [30,51]. Despite widespread validation and use, both instruments have been the subjects of several studies with contradictory results concerning the accuracy of food consumption estimates [30,40,52]. Thus, differences found in this analysis might be due to discrepancies between genders in how they respond to the instruments instead of actual differences in behavior, and caution should be exercised when generalizing these findings to similar cohorts. To reduce population bias, we gave all students an opportunity to be screened to enroll in the program from full campus events. Additionally, this sample was based on participants volunteering for the Get Fruved study who were screened “at risk”; thus, they are not representative of the total college population. Over 50% self-identified as White, females, and consisted primarily of students from large state universities in the Eastern U.S. As such, future exploration should target more diverse populations by factors including equal balances of sex, race, age, and type of institution. Specifically, as this study was primarily to explore sex differences, with the higher frequency of females, caution should be exercised when generalizing to all groups. Likewise, as this study was only cross-sectional, definite associations may not be concluded as longitudinal data analysis of behavioral questionnaires is needed. However, with the large sample of college students analyzed, we were able to find relationships between these behaviors and sex in our study. 

These findings are important to highlight the similar results from previous work in the sex differences of these lifestyle behaviors. However, in this study, we did look at a novel scoring system to address if participants met the criteria for “healthy” ranges of self-reported questionnaires and BMI. In a similar study by Aceijas and colleagues, authors examined similar lifestyle behaviors of university students and found that 60% were not meeting the recommended physical activity, almost 50% had an unbalanced diet, and 30% had low mental health wellbeing. They found women were also less active and that it is important for universities to expand their health promotion programming to include important variables such as these, including mental health [3]. This scoring system, although it identified that males or females may be healthier than each other, assisted us in addressing all lifestyle behaviors and the baseline healthfulness of first-year students attending college. This allowed us to ascertain that most students fall in the middle range of achieving healthy behaviors, with males having a higher proportion of participants with healthier cut-offs than females. 

## 5. Conclusions

This cross-sectional study found differences among lifestyle behaviors among sexes in college students, including self-reported fruit and vegetable intake, percent energy intake from fat, physical activity, perceived stress, and sleep quality. When examining closer, participants were more likely to be classified under the healthy ranges for these tools. Of this self-reported data, males reported higher physical activity, more fruit and vegetable and fat intake, poorer sleep quality, and lesser perceived stress than females. These gender differences should be taken into consideration when designing lifestyle behavior intervention programs for college students. Caution should be exercised when generalizing to all college populations. Further investigation is recommended to better understand the specific relationship between gender and lifestyle behaviors in varied college student populations.

## Figures and Tables

**Table 1:** Demographics and anthropometrics of freshmen students enrolled in Get Fruved by sex.

Variable	Total *n* (%)	Sex		*p*
		Male *n* (%)	Female *n* (%)	
Race/Ethnicity				0.0573
Non-Hispanic White Only	603 (54.2)	198 (32.8)	404 (67.2)	
Non-Hispanic Black Only	117 (10.5)	31 (26.5)	86 (73.5)	
Hispanic/Latino	203 (18.2)	69 (34.0)	134 (66.0)	
Other (including biracial)	190 (17.1)	78 (41.0)	112 (59.0)	
	**Total (Mean ± SD)**	**Male (Mean ± SD)**	**Female (Mean ± SD)**	***p***
Age	18.2 ± 0.5	18.2 ± 0.5	18.1 ± 0.4	0.0096 *

Pearson’s chi-squared tests were used for race/ethnicity. Mann–Whitney U test was used for age. * *p* < 0.05.

**Table 2:** Self-reported anthropometrics of freshmen.

	Total (*n* = 1122)		Male (*n* = 378)		Female (*n* = 744)		*p*
	Median (IQR)	Mean ± SD	Median (IQR)	Mean ± SD	Median (IQR)	Mean ± SD	
Height (cm)	167.6 (12.2)	168.2 ± 8.9	176.1 (9.8)	176.0 ± 7.5	164.3 (9.1)	164.3 ± 6.8	<0.0001 *
Weight (kg)	66.6 (19.7)	69.6 ± 15.8	74.4 (16.8)	76.2 ± 15.0	63.3 (16.9)	65.8 ± 15.2	<0.0001 *
BMI (kg/m^2^)	23.5 (5.7)	24.5 ± 4.8	23.8 (5.7)	24.6 ± 4.3	23.4 (5.6)	24.3 ± 5.1	0.0516

Mann–Whitney’s U test was used for nonparametric continuous anthropometrics variables. Anthropometric data shown in means and standard deviation (SD). Body mass index; BMI. * *p* < 0.05.

**Table 3:** Self-reported behavioral screeners by sex.

	Males (Mean ± SD)	Females (Mean ± SD)	*p*-Value
NCI FV (cup servings/day)	2.79 ± 2.70	2.27 ± 1.88	0.0011 *
NCI fat (% energy)	30.01 ± 2.27	29.34 ± 2.22	<0.0001 *
IPAQ (MET min/week)	3345.70 ± 2358.71	2420.88 ± 1746.22	<0.0001 *
PSS (0–56)	24.68 ± 6.02	27.23 ± 5.91	<0.0001 *
PSQI (0–21)	5.59 ± 2.48	5.95 ± 2.64	0.0254 *

Analysis of covariance (ANCOVA) model controlling for BMI and age used for relationship of behavioral screener scores with sex. NCI F/V: National Cancer Institute fruit and vegetable screener; NCI fat: National Cancer Institute fat screener; PSS: Perceived stress scale; PSQI: Pittsburgh sleep quality index. * *p* < 0.05.

**Table 4:** Healthy range cut-off scores between sexes.

	Total *n* (%)	Male *n* (%)	Female *n* (%)	*p*-Value
Healthy BMI	642 (57.2%)	214 (56.6%)	428 (57.5%)	0.7701
Healthy NCI FV	113 (9.9%)	50 (13.1%)	63 (8.4%)	0.0137 *
Healthy NCI Fat ^^^	1102 (99.0%)	374 (99.2%)	728 (98.9%)	0.7586
Healthy IPAQ	511 (46.8%)	216 (58.5%)	292 (40.8%)	<0.0001 *
Healthy PSS	554 (49.8%)	224 (59.9%)	330 (44.7%)	<0.0001 *
Healthy PSQI	386 (34.5%)	142 (37.3%)	244 (33.1%)	0.1605
Healthy Score ^#^				
1	100 (9.7%)	23 (6.6%)	77 (11.3%)	<0.0001 *
2	277 (26.8%)	69 (19.8%)	208 (30.5%)	
3	311 (30.1%)	107 (30.7%)	204 (29.9%)	
4	233 (22.6%)	100 (28.7%)	133 (19.5%)	
5	99 (9.6%)	44 (12.6%)	55 (8.1%)	
6	12 (1.2%)	6 (1.7%)	6 (0.9%)	

Pearson chi-squared analysis used between healthy vs. unhealthy between sexes. Fisher’s exact test used when cell sizes were low; indicated as ^. For the five tools, participants reaching the ‘healthy’ cut-off range were given a score of 1, and those not reaching the cut-off were given a zero. The five scores were summed to give each participant and individual healthy total score between 0–5. ^#^ One participant classified as a score of zero and, to maintain anonymity, was excluded from analysis. Significant indicated as * *p* < 0.05.
